# Fluid intelligence is related to capacity in memory as well as attention: Evidence from middle childhood and adulthood

**DOI:** 10.1371/journal.pone.0221353

**Published:** 2019-08-22

**Authors:** Aaron Cochrane, Vanessa Simmering, C. Shawn Green

**Affiliations:** 1 Psychology Department, University of Wisconsin–Madison, Madison, Wisconsin, United States of America; 2 ACTNext, ACT, Inc., Iowa City, IA; University of North Carolina at Chapel Hill, UNITED STATES

## Abstract

Human fluid intelligence emerges from the interactions of various cognitive processes.

Although some classic models characterize intelligence as a unitary “general ability,” many distinct lines of research have suggested that it is possible to at least partially decompose intelligence into a set of subsidiary cognitive functions. Much of this work has focused on the relationship between intelligence and working memory, and more specifically between intelligence and the capacity-loading aspects of working memory. These theories focus on domain-general processing capacity limitations, rather than limitations specifically linked to working memory tasks. Performance on other capacity-constrained tasks, even those that have typically been given the label of “attention tasks,” may thus also be related to fluid intelligence. We tested a wide range of attention and working memory tasks in 7- to 9-year-old children and adults, and we used the results of these cognitive measures to predict intelligence scores. In a set of 13 measures we did not observe a single “positive manifold” that would indicate a general-ability understanding of intelligence. Instead, we found that a small number of measures were related to intelligence scores. More specifically, we found two tasks that are typically labeled as “attentional measures”, Multiple Object Tracking and Enumeration, and two tasks that are typically labeled as “working memory” measures, N-back and Spatial Span, were reliably related to intelligence. However, the links between attention and intelligence scores were fully mediated by working memory measures. In contrast, attention scores did not mediate the relations between working memory and intelligence. Furthermore, these patterns were indistinguishable across age groups, indicating a hierarchical cognitive basis of intelligence that is stable from childhood into adulthood.

## Introduction

The construct of fluid intelligence captures the general ability to reason, to flexibly engage with the world, to recognize patterns, and to solve problems in a manner that does not depend upon specific previous knowledge or experience. This construct has been of particular interest within the domain of psychology for a host of reasons, most notably the fact that individual differences in fluid intelligence have been associated with real-world outcomes, including academic and occupational success [1–3]. Indeed, fluid intelligence as a construct originated with the need for educators and employers to assess the aptitudes of their students or employees [4,5].

The psychometric basis of fluid intelligence has also been informed by a growing understanding of its psychological underpinnings. It has long been observed that when participants perform a large battery of cognitive tasks, a dominant latent factor of “general ability” emerges that explains a high degree of individual-level variation on these tasks [6]. In particular, high correlations are ubiquitously found between working memory measures and reasoning measures [7–11]. Working memory refers to the short-term maintenance and manipulation of mental information. The distinctions between working memory and short-term memory have been inconsistent across tasks and theories (e.g., [12–14]. In particular, when relating these processes to fluid intelligence, there is evidence that distinctions between the two constructs may not be wholly clear (see Methods). For consistency, we use the label “working memory” here, although we recognize the potential for dissociation between working memory and short-term memory. These strong correlations between working memory and fluid intelligence have, in turn, led to many distinct lines of research that further probe this relationship. These research tracks include investigations of the utility of working memory over and above fluid intelligence as a predictor of academic success [15] and the possibility of improving working memory and then observing concomitant improvements in fluid intelligence [16].

Researchers have also closely examined the specific aspects of working memory that are most related to fluid intelligence [8]. Results indicate that working memory capacity is predictive of fluid intelligence, implying a general processing-capacity limitation for both categories of tasks [9,10,17]. This area of inquiry is grounded in theories of working memory proposing an interacting set of lower level processes, including the control of attention, that together give rise to short-term maintenance and manipulation of mental representations. Indeed, the most popular formulations of working memory share, at their core, an interaction between memory stores and attention [18,19].

Those theories of working memory that have addressed the role of attention are mirrored by numerous theories of attention that have likewise recognized the role of information maintenance over time. Not surprisingly, many tasks that are putatively assessing “attention” nonetheless have a memory component. Whether intentional (e.g., goal selection) or unintentional (e.g., implicit learning), and whether via a long-term store (e.g., statistical learning) or via a short-term store (e.g., priming), memory representations guide and interact with attentional selection [20–24]. Further, memory for contexts or goals, or even stimuli within a series of presentations, clearly influences the allocation of attention.

Both attention and working memory have limited capacities. The relationship between these two limited-capacity processes is often characterized as being hierarchical, in which low-level perceptual and attentional processing is restricted to a very small amount of information [25,26]. In turn, the capacity of attention constrains the capacity of working memory, which is itself related to fluid intelligence [25,27–29]. Interestingly, the interplay between working memory and attention is arguably also reflected within a number of computational models which do not make a qualitative distinction between memory and attention processes *per se* (e.g., [30,31]). Instead these models can be used to fit behavioral data both from tasks that would typically be associated with “attention” as well tasks that would typically be associated with “memory.” For instance, Ma and Huang [31] compared multiple object tracking performance against simulations of various theoretical models of capacity. Multiple object tracking, in which participants must attend to the identity of some moving targets amidst identical moving distractors, has typically been approached as a test of attention [23,32]; but see [30]. However, various theories of working memory make explicit predictions regarding expected patterns of performance on this task. Ma and Huang [31] treated these models’ predictions as being driven by domain-general capacity constraints, which themselves have been proposed to be the linking process between working memory and fluid intelligence (see, e.g., [17]).

Despite the clear interconnectedness of attention and working memory processes, and between working memory processes and fluid intelligence, the possibility of direct correlations between fluid intelligence and attention have received far less study. Those relations between attention and intelligence that have been reported have largely been focused on attention in the context of canonical "executive function" tasks, rather than canonical attention tasks (e.g., attentional control—[8,27]; behavioral inhibition—[11]). Direct evidence for correlations between visuo-spatial attention measures and fluid intelligence is rarer (with the notable exception of a large literature on intelligence and processing speed, which could potentially be framed in terms of attention, e.g., [33–35]. One goal of the present work is to examine whether similar correlations are observed between canonical visuo-spatial attention tasks and fluid intelligence tasks, and, if so, whether the underlying mechanisms appear similar as those that have been shown to link working memory tasks with fluid intelligence tasks.

### Developmental comparisons

Beyond the high-level question above, there is also significant interest in the extent to which cognitive functions and their interrelations shift through development [36]. Our understanding of cognition is inextricably linked to an understanding of development [37]; in order to understand the relations between specific abilities and behavioral tasks (as well as other measures that can clarify them), we should ensure that we are able to model the changing nature of this over the lifespan [36,38–40].

Developmental change from childhood to adulthood is associated with quantitative improvements in processes as diverse as reading, reasoning, motor skills, and working memory. Despite the ubiquity of quantitative improvements, evidence for qualitative changes in patterns of performance is more equivocal (e.g., in terms of the way that performance on different tasks correlate/load together). While there is some evidence suggesting that certain patterns may shift through development [27,41], the stability of cross-construct patterns of performance between age groups has provided the foundation for influential theories of cognition and intelligence (e.g., [38]**)**.

If correlations between abilities are present across individuals within age groups, the most parsimonious expectation is that similar correlations would be present in other age groups. Age-related differences in the interrelations of cognitive abilities would precipitate a need for a mechanism of age-related change not only in the component processes but also in how they relate. On the contrary, the null hypotheses (i.e., stability across development) does not require a mechanism of change for the relation, only the shared component process. This stability in the structure of cognition across ages has been observed in previous longitudinal studies in childhood (e.g., [15]). If each individual’s cognitive abilities have stable interrelations over development, we expect cross-sectional results of the structure of cognition to likewise be similar in both children and adulthood.

### Current work

Given existing computational and developmental accounts of cognition, there remains a need to examine the interrelations between measures of working memory, attention, and intelligence. Here we test the relations between performance on a variety of basic psychological tasks and Raven’s Progressive Matrices (a common measure of fluid intelligence) in both children and adults. We compare relations between cognitive abilities in middle childhood (~ 8 years old) with those in early adulthood by measuring performance on 13 computerized tasks. These tasks ranged from simple visuo-spatial attention tasks to measures of vocabulary and working memory span.

Correlations have been observed in previous work between some of these measures, such as between fluid intelligence and working memory tasks (e.g., N-back, spatial span). In the case of the N-back task, this relationship appears based upon the strong capacity-loading dimension [17]. In other words, there are many ways one can potentially alter the difficulty of the N-back task (e.g., altering presentation speed, altering the prevalence of recently seen items, etc.), yet differences in how performance changes as a function of load (i.e., “N” in the case of N-back) is most strongly related to differences in measured intelligence. We expect to replicate these findings in both children and adults. Following Ma and Huang [24], one specific novel interest here is the relation between performance on multiple object tracking (MOT) and fluid intelligence. Although this relation has not been previously explored, a theoretical link between capacity-loading tasks (which MOT is, despite typically being considered an attention task) and fluid intelligence suggest that a significant relation will be found.

Given the full set of measurements from the task battery, our first goal was to identify the degree to which the measures are generally related to fluid intelligence and the degree to which these relations are stable across development. Although consistently positive cross-task correlations are cited as evidence for a common latent general ability, our battery was explicitly designed to test independent aspects of visual attention and working memory. Thus, our expectation was that some, but not all the tasks in the battery would be related to fluid intelligence. Our second goal was to test the conventional separation of cognitive tasks into *attention* and *memory* categories. Although these literatures are often distinct, theories regarding the link between processing capacity and fluid intelligence suggest that tasks with a significant capacity loading should correlate with fluid intelligence (even if that task is typically considered an attentional task). Here we use the differential relatedness to reasoning scores found in the previous analyses to specify model comparisons using Bayesian regression and mediation. We use these models to quantify the moderating role of age and the mediating role of attention or memory.

## Method

### Participants

This study was approved by the University of Wisconsin-Madison Education and Social/Behavioral Sciences Institutional Review Board (#2014–0283). Written consent was obtained from each adult participant, and from a parent or legal guardian of each child participant. The final reported sample includes 42 children (age range = 7.1–9.2 years, *M* = 7.9, SD = .57; 19 females) and 80 young adults (age range = 18.0–23.1 years, *M* = 19.3, SD = 1.03; 58 females). Individual demographic data were not collected from these families, but the community from which we sampled is primarily white, non-Hispanic, middle- to upper-middle-class, and monolingual English-speaking. An additional 2 children and 12 adults participated but were excluded based upon performance that indicated either task misunderstandings or some other form of non-compliance (see S1 File for information on exclusions). Child participants were recruited from a university participant database of parent volunteers from a small city in the Midwestern United States. Young adult participants were recruited from Introduction to Psychology courses at a large public university. Participants were not screened for language proficiency. Families of child participants received $40 for their participation. Adults received course credit for their participation.

### Apparatus and procedure

All tasks were programmed in MATLAB using the Psychophysics Toolbox [42,43]. Tasks were presented on a 22-inch Dell widescreen monitor by a Dell Optiplex computer running Windows 7, at approximately 60 centimeters of viewing distance. Stimulus details are included below within the description of each task.

Participants completed 13 tasks in a pre-specified order (Attention Network Task, Change Detection, Change Detection with Filtering [CD filter], Orientation Delayed Match to Sample, N-back, Peabody Picture Vocabulary Test, Spatial Span, day break for children, Position Delayed Match to Sample, Color Delayed Match to Sample, Useful Field of View, Multiple Object Tracking, Raven’s Progressive Matrices, Enumeration; see details below). The task order was chosen to vary the cognitive demands of temporally adjacent tasks and reduce the potential for cognitive fatigue. Child participants completed the tasks in two 2-hr sessions across different days while adult participants completed the tasks in one 2-hr session. All tasks had short (approximately 5-trial) practice components consisting of very easy trials. Before each task, an experimenter read the instructions for the task to child participants, then presented the practice trials and answered any questions the child had. After the experimenter judged that the child understood the task instructions, he or she began the test trials. Instructions were presented in writing at the beginning of each task for adults, who then initiated the task when they were ready to proceed. Participants were given the opportunity to ask questions and to take breaks between or during tasks.

### Selection of tasks for the battery

Our battery included tasks intended to have a wide variety of cognitive processing demands. These included tasks canonically related to reasoning scores, such as N-back, as well as tasks that were less likely to be related to reasoning scores. The inclusion of tasks known to be related to reasoning scores allows for confirmation of the reliability of our measures and estimation of informal upper bounds of our expected cross-task associations. The inclusion of tasks unlikely to be related to reasoning scores served several important purposes. For instance, it allowed us the opportunity to examine a number of potentially subtler inter-relations between processes inherent in our tasks of interest and fluid intelligence. As a possible example, if a positive relation was observed between MOT and fluid intelligence, some of these additional attention or memory tasks could allow us to test mediations regarding what sub-processes might underlie the relation.

Further, the tasks included in the overall battery together represent a reasonable cross-section of visuospatial processing tasks, providing “coverage” of alternative hypotheses regarding individual differences. For example, the presence of tasks that do not significantly correlate with fluid intelligence is necessary in order to demonstrate that any positive relations that are observed (e.g., between MOT and fluid intelligence), are process-specific. We tested this process-specificity with the first set of analyses focusing on the possible presence of widespread positive correlations (a “positive manifold”; [6,36]). Such positive manifold is often observed in cognitive batteries, particularly when the battery is constructed to include only tasks that one might *a priori* predict would be related to fluid intelligence. However, given the composition of our battery, the expectation is instead to observe large variation in the strengths of correlations between individual cognitive measures and intelligence.

#### Visuo-spatial working memory measures

These tasks were designed to test visual working memory at varying levels of precision and capacity. None of these involve classic dual-task (i.e., “maintenance” plus “manipulation”) working memory tasks due to the relative difficulty of implementing these complex tasks in child populations. However, at high difficulty levels, previous work suggests a commonality between dual- and single-tasks’ relations to matrix reasoning measures [17,44]. Indeed, in populations of children and older adults, single-task spans are often utilized as measures of working memory (e.g., [45–48]).

**Fig 1:**
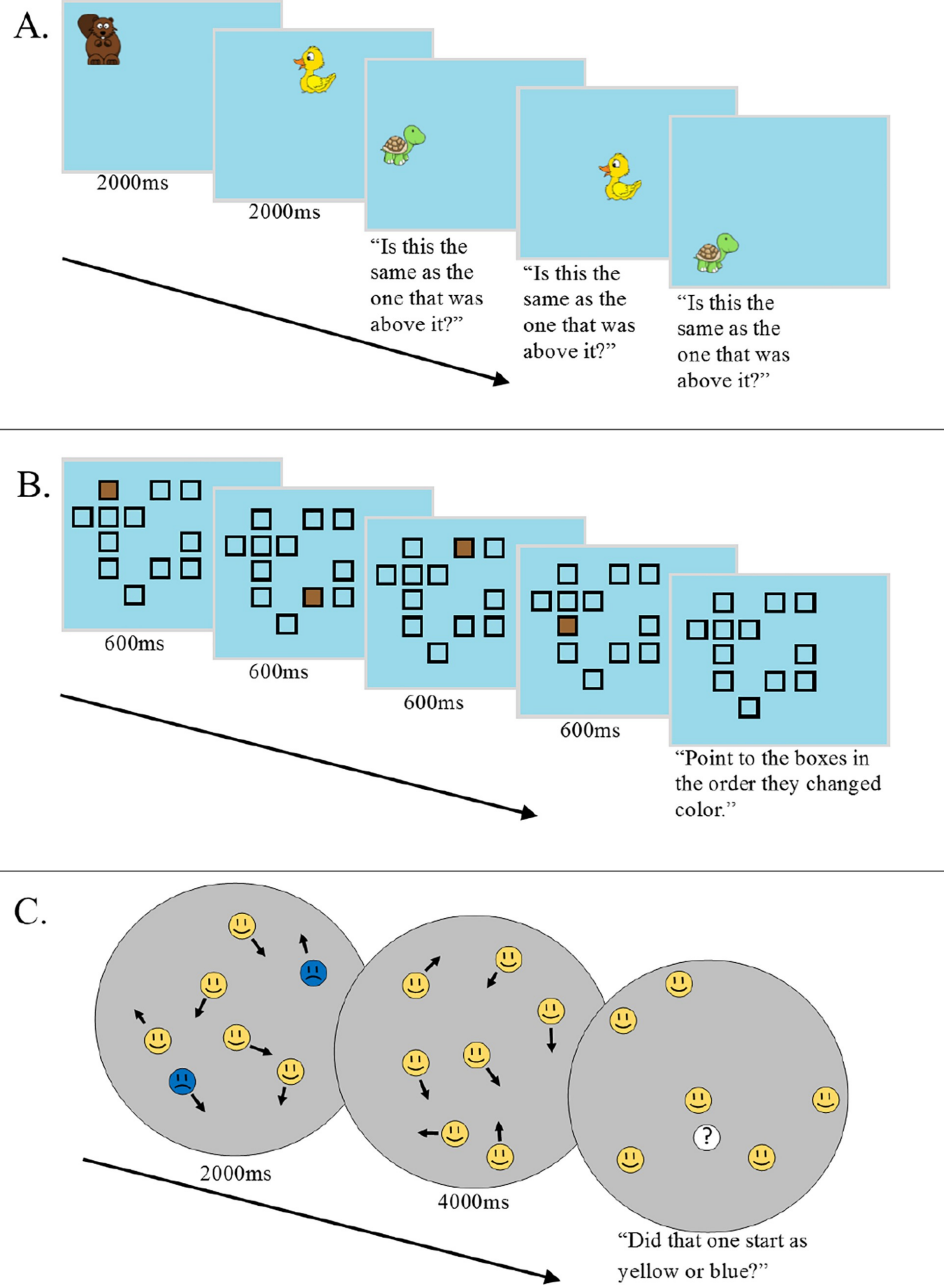
Examples of tasks. Examples of 3 tasks. All parameters remained the same for both children and adults except set size. Children were given the verbal prompts shown here, while adults were given instructions prior to the tasks. (A) In the N-back task, a series of cartoon animals were presented in an invisible grid. Participants responded whether the item that had appeared above the current item was the same or different than the current item. Set size (i.e., ‘N’) was identical to the number of columns. (B) In the Spatial Span task, participants saw a series of items filled in within a randomly-generated set of squares. They then reproduced the sequence they saw. (C) In Multiple Object Tracking (MOT), participants tracked several blue target faces moving amidst many yellow distractor faces. The targets then became identical to the distractors and all items continued to move. After 4 seconds of motion, one item was cued and the participant had to identify whether the cued item started as a blue or yellow face.

1] N-Back task (see Fig 1A): The N-back task is a common running span measure in which a series of stimuli are presented and the participant has to compare the current stimulus to the stimulus that was presented N items prior; for example, on 2-back trials, participants are required to compare the identity of the third item to the first item, the fourth item to the second item, the fifth item to the third item, and so on [49]. We utilized a variation of the standard N-back working memory task that has previously been used to assess differences across distinct groups or across the lifespan [50–52]. Minor modifications were made to the standard design to ensure the task was easily understood by children. In this task, the screen space was evenly divided into 7 columns from left to right, and 21 rows from top to bottom. Although the column/row divisions were not explicitly displayed on the screen, the participants were made aware of this fact during the task explanation and this setup aided participants in keeping track of the temporal position of items [50]. Each trial began by indicating the number of columns that were going to be used on that trial (the ‘N’ in the N-back–i.e., on a 2-back trial, 2 columns would be used; see Fig 1]; this allowed the vertical spatial alignment of items to serve as a cue for which items should be compared. For ease of exposition, we will briefly describe a 2-back trial–with the other levels of ‘N’ being simple extrapolations of this base description.

Participants pressed a key to begin each run. After 1 s, a cartoon animal was presented on a blue background in the first column of the first row. All the animals were visually distinct and presented in nameable different colors (brown beaver, blue fish, red crawfish, tan clam, yellow duck, green turtle) and subtended approximately 1.3° of visual angle. Then, after 2 s, the first animal disappeared and a new animal appeared in the second column of the first row. After a 2 s delay, the second animal disappeared and an animal was presented in the first column of the second row. The participant then indicated via a keypress whether this animal matched the one that had appeared directly above it (i.e., the animal that had been presented in the first column of the first row, or 2-back). After the participant entered a response, the third animal disappeared and an animal appeared in the next location (second column of the second row), with the response now indicating whether the animal matched that which had been presented in the second column of the first row. The next animal appeared in the first column of the third row, and so on until the participant had made a total of 20 responses. The other values of “N” progressed in the same manner across N number of columns. Adults completed set sizes (“N”) 1 through 7 followed by 7 through 1 [20 trials per run), while children completed set sizes 1 through 5 followed by 5 through 1 (15 trials per run). Overall percent correct was then calculated from all trials.

2] Spatial span task (see Fig 1B): The spatial span task that was utilized was a variant of the Corsi block-tapping task, a classic measure of visuospatial short-term memory [53–56]. Each trial began with the presentation of an array of outlined squares (each subtended 1.25° visual angle) in 12 locations selected pseudo-randomly from a virtual 5 x 5 grid of possible locations on a blue (RGB = 153, 217, 234) background. Then, a subset of these squares (corresponding to set size) were briefly filled in, one at a time, for 0.6 s before turning back to unfilled. After the final square changed back to unfilled, the participant was prompted to use the mouse to click on the squares in the order that they had changed color (with the squares changing color when clicked to verify each choice). Children completed 25 trials consisting of 5 each of set sizes 2 through 6. Adults completed 20 trials consisting of 4 each of set sizes 4 through 8. In both age groups, set sizes were randomly shuffled and intermixed.

The dependent variable for this task was calculated using two criteria. The ‘strict’ criterion credited only exact location/temporal matches between the presentation and response (i.e., a response out of order was considered incorrect). The ‘lax’ criterion credited responses that corresponded to any item from the presentation, regardless of order. This measure recognizes that, for example, a participant may forget the first item but then reproduce the sequence accurately starting with the second item. Such a response would result in a score of 0 using the strict criterion, but clearly indicates some memory for the array. For all statistics, we used the average (per trial) between strict and lax scores; however, we note that the results are not qualitatively different when using only strict or lax scores.

3] Visual array change detection (CD): One-shot visual change detection involves the sequential presentation of two arrays of colored items, with the participant’s task being to detect the presence of a change between the two arrays [25,57,58]. Here stimuli were cartoon fish, subtending 1.8° of visual angle wide and separated by at least 0.9° on a blue background, drawn without replacement from the following colors (*R*,*G*,*B)*: white [2*36*, *236*, *236]*, red [2*55*, *0*, *4]*, purple [1*36*, *12*, *146]*, orange [2*55*, *127*, *39]*, green *[98*, *249*, *44]*, blue *[0*, *35*, *255]*, yellow [2*55*, *255*, *0]*, brown [1*13*, *56*, *0]*. Stimuli were randomly placed at locations in a virtual grid (adults: 5 x 5, children: 4 x 4] and presented for 250 ms immediately after a brief auditory cue. All stimuli then disappeared for a 1-s delay, after which a test array was presented until the participant responded. The test array was either identical to the first array, in which case participants were instructed to press a key labeled “same,” or one item changed color, in which case participants were instructed to press a key labeled “different.” Children completed 4 “different” trials and 4 “same” trials each for set sizes 2, 3, 4, 5, and 6, and adults completed 6 “different” and 6 “same” trials each for set sizes 2 through 7. Trial order was randomized. Overall percent correct was calculated and used in all analyses; *A’* measures were also calculated [59] but the results were not qualitatively different than percent correct, so we report the simpler measure (percent correct) here.

4] Change detection task with distracting irrelevant items (“CD filter” task): This was based on the change detection task described above, but manipulated the number of distracting items rather than target items [25,60]. The target set size was fixed at 3 for children and 4 for adults on all trials. In addition to the colored cartoon fish targets on the screen there were circles with the same dimensions as the fish. These circles were described as “rocks” to participants, and were drawn from a non-overlapping color set from the fish. Adults completed 80 trials and children completed 60 trials, which were evenly divided between high-distractor (adults = 10, children = 6] and low-distractor (adults = 3, children = 2) condition. The dependent measure was the percent correct on low-distractor trials minus the percent correct on the high-distractor trials, indicating a decrement in change detection ability with additional task-irrelevant distractors.

5*a-c*) Delayed Match to Sample (position, color, orientation): Three separate visual short-term memory precision tasks were completed (memory for color, position, and full-contrast Gabor orientation respectively; see [61]). In the position memory task, participants saw a circle on the screen for 1000 ms, which was followed by a blank screen. After a delay of 500 ms, an identical circle appeared offset 0, .2, .7, and 1.5 degrees to the right or left. Participants pressed arrow keys to identify the direction of offset, and completed 80 of these trials. The dependent variable in this task was a 79% threshold calculated from a logistic fit to responses. The color and orientation tasks followed the general procedure of [61], with timing identical to the position memory task. In these two memory tasks, the post-delay stimulus could be the same or different than the original stimulus; if different, the difference was defined by either an orientation offset or an offset in a standardized 360-degree color space (see [61]). Offsets increased when participants responded that the stimuli were the same and decreased when participants responded that the stimuli were different, according to the procedure in [61]. The dependent variable on these tasks was the mean of the smallest color space distances for which participants responded that the colors were different.

#### Attention measures

1] Visual enumeration task: The visual enumeration task required participants to identify the number of stimuli that were briefly presented. While many foundational applications of this paradigm have emphasized the speed with which participants could identify the correct number of items (e.g., [62–65]), we were instead interested in participants' ability to correctly identify the number of items after a short presentation [66]. On each trial, a number of yellow cartoon fish (modeled after [67] were presented in random locations within a virtual 5x5 grid in the middle of the screen for 250 ms (children) or 150 ms (adults) on a blue background. Each fish stimulus was 1.9° of visual angle wide, and at least 0.95° of visual angle separated each stimulus. After stimulus presentation, participants responded by typing the number of fish they saw using the number keys on a standard keyboard. All participants completed four trials per set size (one through ten, randomly intermixed), and the percent correct for all trials was used as the aggregate performance score.

2] Multiple Object Tracking (MOT; see Fig 1C): The MOT task involves attending to a number of moving targets amidst identical distractors [23]. The version employed here was a child-friendly version of the task similar to that which has been described previously [68]. Fourteen cartoon faces (each subtending .8° of visual angle) for child participants, or sixteen cartoon faces for adult participants, were presented inside a gray circle on the screen that subtended 10° of visual angle from its center. All faces were yellow circles with black lines depicting a ‘happy face’, except for a variable number of faces, which were blue circles with black lines depicting a ‘sad’ face. The blue ‘sad’ faces were identified as the targets that the participant should track (and thus the yellow ‘happy’ faces were distractors). Each trial consisted of all the cartoon faces moving within the gray circle at a speed of 5 deg/s, with direction of movement determined stochastically. The faces never overlapped or touched, and were programmed to ‘bounce’ off each other and the walls of the gray circle. After 2 s, the blue target faces changed to match the yellow, happy distractor faces. After an additional 4 s of movement, the faces halted and a white circle containing a question mark replaced one of the faces. The question mark had a 50% chance of appearing over a target face. Participants were required to indicate whether the indicated face had been a blue target face at the onset of the trial. Children were tested on 10 trials each of set sizes 1, 2, 3, and 4 targets. Adults were tested on 5 trials of set size 1 and 10 trials each of set sizes 2, 3, and 4, and 5. These set sizes were chosen to ensure that no participants would perform at chance, due to tracking 1 item being very easy, but capacity-related individual differences would be evident at higher set sizes. Trial order was fully randomized. Overall percent correct was calculated and used in all analyses.

3] The Attentional Network Task (ANT; [67,69]: The ANT is a measure of several dimensions of visual attention involving a directional response to an oriented central stimulus that is flanked by response-compatible or response-incompatible stimuli. These central and flanking stimuli are presented in combinations of location cues as well as response-compatible and response-incompatible distractors. Subtracted mean response times (e.g., incompatible-distractor trials minus compatible-distractor trials), normalized by individual participants’ overall mean response time, are intended to index attentional orienting, alerting and conflict resolution. We utilized a 96-trial version of the task adapted from [56] using yellow fish as the directional stimuli. All task parameters (e.g., size of stimuli) were modeled directly from [56].

4] The Useful Field of View (UFOV) visual search task: The UFOV task [70] is designed to test individuals’ ability to identify briefly-presented peripheral information. In this task participants see a display of items (white squares) arranged in two concentric circles and along 8 lines radiating from the center of the screen. The participant must identify the line on which an oddball item (a white star) occurred. A concurrent task, designed to encourage central fixation and ensure the peripheral nature of the main task, involved an identification of a central stimulus, a small cartoon face, as having long hair or short hair. Adults and children each completed 50 trials of a 3 down/1 up adaptive staircase. The staircase procedure converges on a threshold value; these threshold values were log-transformed to approximate normality then sign-flipped to make better performance be associated with higher values for consistency with other tasks.

#### Intelligence measures

1] Raven's Progressive Matrices were utilized as our measure of fluid intelligence. These matrices are a measure of reasoning commonly used in cognitive training paradigms (e.g., [71–73] as well as cross-sectional studies of the cognitive bases of reasoning [74]. Despite the fairly small number of trials, Raven’s Matrices have long been the gold standard for reasoning measures. Nonetheless, our use of only one measure of this construct does limit the conclusions we can draw.

In this task, a series of pattern completion problems are presented to the participant. The rules for the patterns (e.g., perceptual matching, mutual exclusivity) become more difficult as items progress later in the series. We presented a selection of 4 items from set A, 6 items each from sets B, C and D, and 4 items from set E of Raven's Standard Progressive Matrices to children (26 items total), and even-numbered items from Raven's Advanced Progressive Matrices to adults (18 items total). All participants were limited to 10 minutes in completing the task, although as they completed items they were unaware of the amount of time remaining. The adults’ selection was patterned from previous cognitive training work that divided reasoning tests into two halves for pre- and post-tests [17,71], while the children's selection was designed to maximize individual differences by presenting a large range of difficulty in stimuli in a relatively brief amount of time. Our outcome measure for this task was percent correct of the total items.

2] As a test of verbal knowledge that could covary with Raven’s scores (particularly in children), each participant completed a non-progressive selection of items from the Peabody Picture Vocabulary Test (PPVT; [75], a standardized 4-alteranative-choice test of vocabulary. Stimuli were presented on the full screen and a recording was played directing the participant to choose a certain cartoon picture (e.g., the participant would see 4 pictures on the screen and would hear a phrase like “point to cupola”). Children were provided the opportunity to ask the experimenter to repeat the word if they desired. Children completed items 109–156 while adults completed items 169–216.

## Results

Univariate and bivariate descriptive statistics are reported in S2 File.

### Full-dataset results

#### Analytical approach

We first used the full covariance structure of our data to test for the presence of a positive manifold. We examined the magnitudes of bivariate correlations as well as the degree to which our data could be explained by a single underlying latent variable. These two methods provided evidence regarding the hypothesis that a positive manifold in task performance was present. If correlations between tasks were uniformly large and positive, and if the majority of variation could be explained with a single latent variable, that would be evidence for the classic general-ability definition of fluid intelligence [6,39]

In contrast, if cognitive tasks were not generally positively correlated with Raven’s scores, this would be evidence for some degree of process-specificity of reasoning (i.e., that reasoning is not reliably related to all possible tasks–which was our a priori assumption). Indeed, our methods involved the intentional inclusion of a wide variety of cognitive tasks which themselves were designed to demand a variety of processes. Process-level heterogeneity by design should lead to a performance covariance structure that is likewise heterogeneous, and we did not expect a strong positive manifold or single-component latent variable structure. However, our analyses in this section also provide initial evidence for which tasks that are most related to our measure of reasoning, Raven’s. We end this section by examining the evidence for a subset of reasoning-related tasks, which can then inform more specific model comparisons in the next section.

#### Bivariate correlations

As an initial measure of a positive manifold, we calculated the proportion of all pairwise correlations that were higher than a series of possible thresholds. We did this by resampling our dataset 10,000 times with replacement and calculating the percent of above-threshold product-moment correlations for each threshold. Task scores were sign-flipped, if necessary, so that larger values would indicate better performance in these analyses.

**Fig 2:**
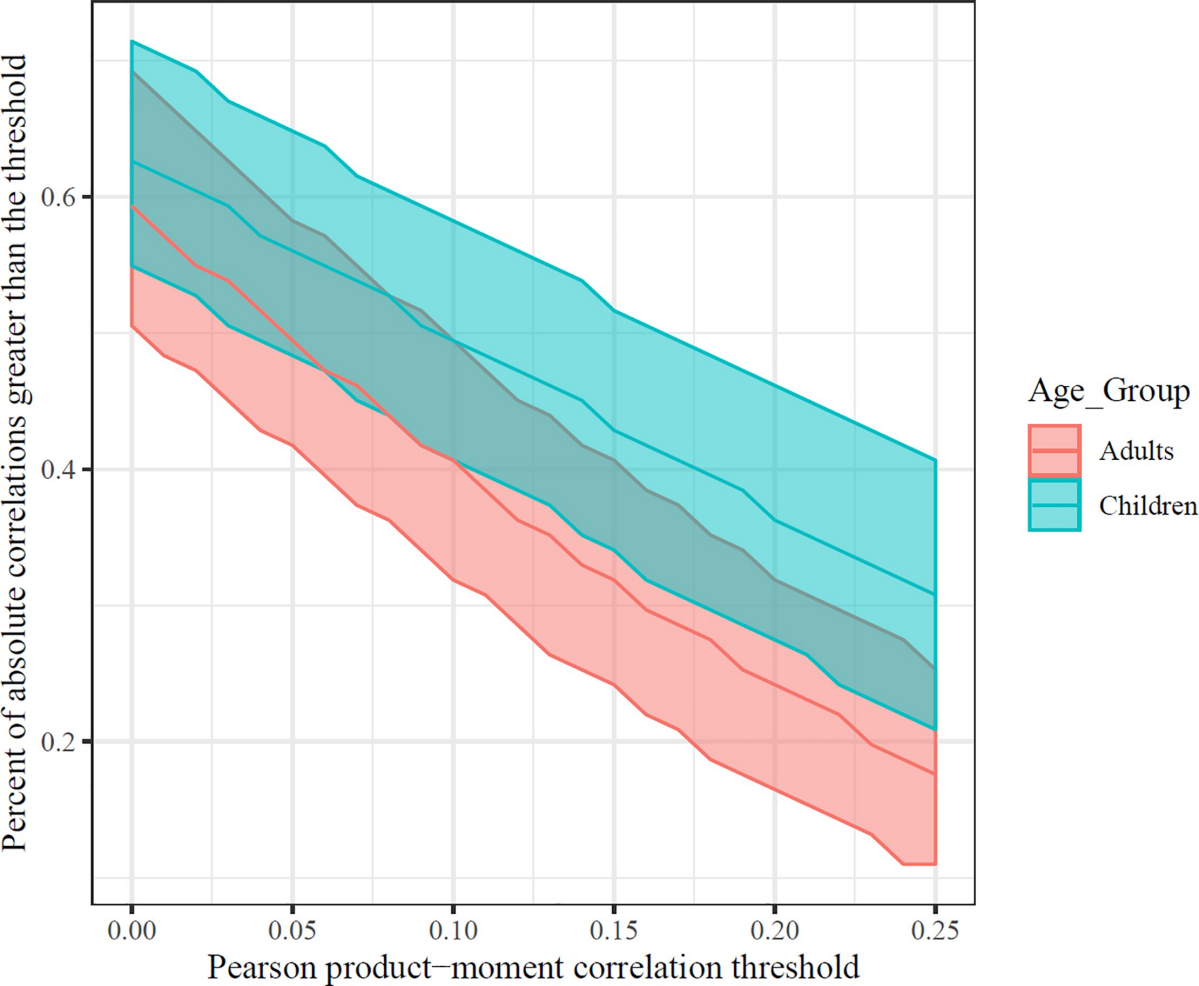
Test of positive manifold in children and adults. As the threshold for identifying a “positive manifold” increased, the number of correlations that satisfy that threshold decreased. Error bands denote 95% percentiles from 10,000 bootstrapped samples for each of 26 evenly spaced correlation thresholds. Thresholds for statistical significance, at alpha = .05, are r = 0.304 (children) and r = 0.219 (adults).

When this threshold was restricted to only correlations that were positive (i.e., > 0), a slight majority of correlations appeared to demonstrate a positive manifold (see Fig 2). In contrast, if a threshold was introduced such that tasks must share just 1% of their variance (i.e., correlation at or above 0.1), then fewer than 50% of correlations satisfied the expectation of a positive manifold. Bootstrapped Spearman rank-order correlations followed the same qualitative pattern (see S3 File). Since there was no clear positive manifold, with many very small correlations in our data, it was more likely that certain key tasks should be the focus of our comparisons here. Indeed, only a sub-set of measures appeared to be related to domain-general fluid intelligence ability. This was the expected result, in that some tasks were chosen for the battery that were unlikely to load particularly strongly on fluid intelligence abilities.

#### Latent variable decompositions

In order to confirm the lack of a positive manifold found above we conducted a second analysis to test the extent to which a single factor could explain performance across all tasks in the battery. To this end, we examined the components produced by an eigenvalue decomposition of the correlation matrices when excluding Raven’s (i.e., Principal Components Analysis; PCA). This analysis was able to provide evidence supporting our observed lack of a positive manifold. In addition, we used this analysis to identify certain tasks most likely to be related to fluid intelligence.

As was true in the general patterns seen in the bivariate correlation matrices, PCA also did not support a single-component latent structure. In the dataset including both children and adults and looking only at tasks other than Raven’s, 5 eigenvalues were above 1, with 2 being above the parallel-analysis cutoffs (actual eigenvalues: 2.96, 1.44). Yet, while 2 components survive the parallel analysis cutoff, we feel caution is warranted in interpreting these components [76,77]. For instance, when splitting the data by age group, only one component in each age group survived a parallel-analysis component comparison. This implied a possible dissociation between adults’ and children’s patterns of cognitive correlates of intelligence. Age-related differences between the tasks associated with high first-component loadings would provide evidence for developmental changes in the relations of cognitive abilities. Below, we examine each age group’s first-component loadings in order to understand these patterns in more detail.

#### Identifying target cognitive tasks

Examining the first component in each age group, it was clear that tasks with high working memory demands had consistently large loadings (see Table 1). In addition, several attentional tasks had high loadings as well, with enumeration loading the most strongly onto this first component.

**Table 1:** PCA component-1 task loadings for each age group.

	Adult Component 1	Child Component 1	Difference in Loadings
Enum.	0.719	0.803	-0.084
Spat.Span	0.715	0.740	-0.026
N-back	0.684	0.686	-0.002
UFOV	0.629	0.491	0.138
MOT	0.546	0.659	-0.114
PDMS	0.486	0.457	0.029
CD	0.451	0.466	-0.015
PPVT	0.156	0.419	-0.263
ANT alert	-0.016	0.320	-0.337
ANT conflict	-0.100	-0.219	0.118
CD filter	-0.140	-0.344	0.204
CDMS	-0.255	-0.344	0.089
ODMS	-0.442	0.141	-0.583

Tasks are sorted according to adults’ loadings. Children’s loadings follow a qualitatively similar pattern. Abbreviations: Enum = Enumeration; Spat.Span = Spatial Span; UFOV = Useful Field of View; MOT = Multiple Object Tracking; PDMS = Position Delayed Match-to-Sample; CD = Change Detection; PPVT = Peabody Picture Vocabulary Test; ANT alert = Attention Network Task alerting score; ANT conflict = Attention Network Task conflict score; CD filter = Change detection with distracting items; CDMS = Color Delayed Match-to-Sample; ODMS = Orientation Delayed Match-to-Sample

The construct of fluid intelligence describes a latent general ability to perform on tasks, particularly novel ones on which prior knowledge would not help. The tasks in this battery overwhelmingly fit this description (apart from PPVT, which is specifically designed to test prior knowledge, a central aspect of crystallized intelligence). As such, the general component just described should be related to a general performance factor such as fluid intelligence. Further evidence may be derived from an examination of bivariate correlations. Specifically, we were interested in whether correlations between cognitive measures and Raven’s scores were high for the same tasks with high component-1 loadings. Further, differences between children’s and adults’ correlations would provide initial evidence for development-related qualitative differences in the cognitive correlates of reasoning. To test this, we examined the correlations between each task and Raven’s scores (see Table 2; see S2 File for the full correlation matrices). In particular, we compared these correlations to the patterns seen in the component analysis reported above, and we statistically test the differences between children’s and adults’ correlations.

**Table 2:** Correlations of cognitive tasks with Raven’s, separated by age group.

	PCA rank	Adult Raven’s correlations	Child Raven’s correlations	Difference in correlations	Difference p-value
Spat.Span	2	0.412	0.414	-0.001	0.994
N-back	3	0.383	0.298	0.085	0.624
Enum.	1	0.214	0.313	-0.098	0.590
MOT	5	0.167	0.377	-0.210	0.247
PDMS	6	0.123	0.139	-0.016	0.932
ANT alert	9	0.112	0.239	-0.126	0.507
UFOV	4	0.087	0.154	-0.067	0.728
CD	7	0.051	0.326	-0.274	0.145
PPVT	8	0.040	0.603	-0.563	0.001
ANT conflict	10	0.026	-0.171	0.198	0.311
ODMS	13	-0.020	0.066	-0.086	0.660
CD filter	11	-0.050	-0.123	0.074	0.705
CDMS	12	-0.057	-0.296	0.239	0.207

Tasks are sorted according adults’ correlations. “PCA rank” refers to the ordering, from Table 1, of adults' first-component task loadings. Thresholds for statistical significance, at alpha = .05, are r = 0.304 (children) and r = 0.219 (adults). Abbreviations: Spat.Span = Spatial Span; Enum = Enumeration; MOT = Multiple Object Tracking; PDMS = Position Delayed Match-to-Sample; ANT alert = Attention Network Task alerting score; UFOV = Useful Field of View; CD = Change Detection; PPVT = Peabody Picture Vocabulary Test; ANT conflict = Attention Network Task conflict score; ODMS = Orientation Delayed Match-to-Sample; CD filter = Change detection with distracting items; CDMS = Color Delayed Match-to-Sample

As Table 2 shows, the tasks with the highest correlations with Raven’s generally had the highest loadings on the common component. These included spatial span, N-back, enumeration, and MOT. Despite this qualitative pattern of similarity, neither the enumeration nor the MOT correlations reached conventional statistical significance in adults. The correlations between these four tasks generally were similar between age groups, while certain other correlations are quite different. Most notably, the correlation of PPVT scores and Raven’s scores was significantly higher in children than adults (*p* < .01).

Of these four top tasks that were both correlated with Raven’s and loaded highly onto the common latent component, two were canonical memory tasks (i.e., spatial span and N-back) and two were canonical attention tasks (i.e., enumeration and MOT; note that UFOV had relatively high component loadings while having weak correlations with Raven’s). This provided evidence that the relations between cognitive tasks and intelligence measures are not divisible into strictly *memory* or *attention* categories, but specific processing demands of tasks may be related to measured intelligence. Further analyses explored these tasks in greater detail.

### Testing specific relations between reasoning and cognitive measures

The preceding analyses, including Pearson product-moment correlations and PCAs associated with these correlations, provided an exploratory sense of the relations between cognitive measures in our battery as well as developmental differences. The overall covariance structure of cognitive performance did not indicate a positive manifold nor strong developmental changes in the structure of cognition.

We next fit specific regression models, allowing for more specific characterizations of the relations between age and cognition. In this section, we report generalized linear models which allowed for tests of the reliability of cognitive measures in predicting Raven’s scores. In these models, interactions with age provide evidence regarding the presence or absence of age-related changes in the cognitive correlates of intelligence. We tested specific tasks, informed by the previous analyses, as well as composites of these tasks categorized as *attention* or *working memory*. Given reliable results, we next tested the uniqueness of these cognitive correlates of intelligence. Finally, we used mediation models to assess the degree to which attention or memory measures predict Raven’s scores over and above the other category of task.

#### Analytical approach

Each of the following analyses was the output of one Bayesian regression using the *brms* package in R [78]. A beta response distribution was used because Raven’s accuracy scores are bounded between 0 and 1, and for interpretability coefficients are reported on log-odds scales. Results are reported here in terms of two numbers, the regression coefficient (i.e., expected value [*b*]) and the proportion of the posterior distribution with the same sign as that expected value (congruent density [*cD*], e.g, a positive expected value is paired with the percent of posterior samples that were positive). This provides a simple and intuitive sense of the probability of the hypothesis that a given parameter is non-zero. We adopt a threshold value of *cD* = 0.975, roughly corresponding to a two-tailed frequentist *alpha* = 0.05. Note that the main effect of age is not reported in this section because it is likely to be a theoretically-unimportant artifact of task-difficulty differences across ages. More detailed information about each model’s results can be found in tables in S3 File.

Each of these regressions sampled 5 chains for 20,000 samples each, with the first 10,000 of each chain’s samples being discarded as warm-up. A thinning interval of 2 was implemented. Default priors from *brms* were used in each model. In every case, the precision parameter *phi* was estimated as a random effect that varied by age, thereby controlling for age-related distributional differences in Raven’s scores. All models converged with r-hat values below 1.02, indicating a very small potential scale reduction factor across chains.

In S3 File frequentist fits of these models are reported. Also, reported there are bootstrapped frequentist estimates of parameters from models which randomly sample an equal number of children and adults, thereby mitigating possible bias in parameter estimates due to unequal sample sizes (see S3 File).

#### Predicting reasoning using measures of visuospatial working memory

**Fig 3:**
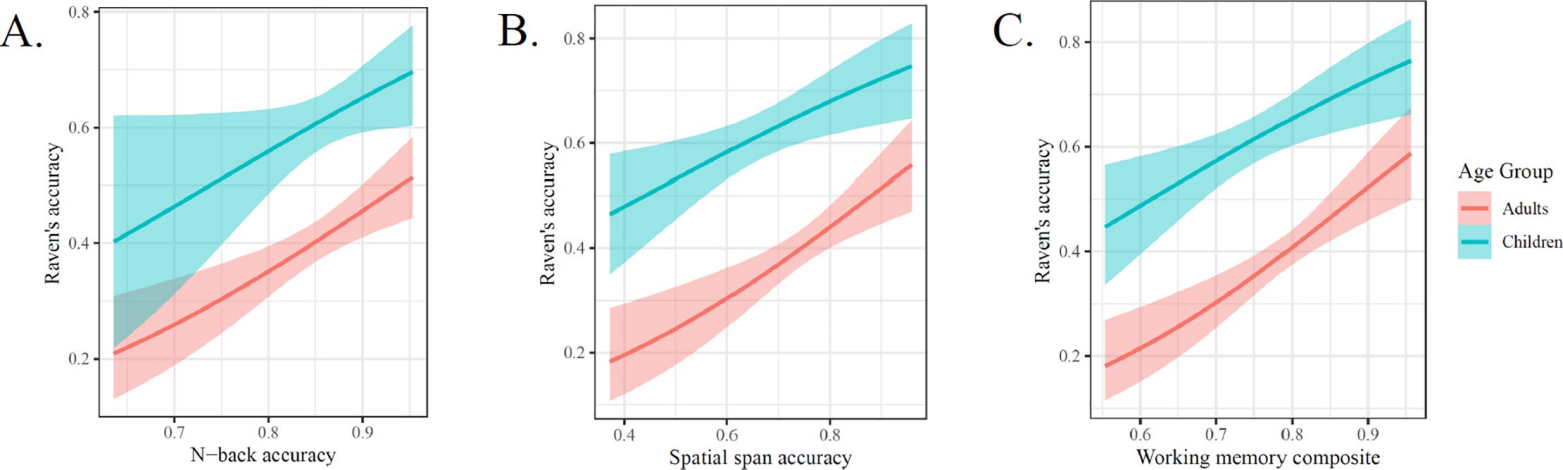
Effects of key working memory measures in predicting Raven’s scores. Main effects of each memory measure were reliably predictive of reasoning scores. No interactions with age were reliable.

We start by looking at relations between working memory measures and Raven’s as moderated by age (see Fig 3). Given the lack of age-related differences in the previous analyses, despite our expectation of reliable main effects of cognitive predictors, we did not expect any reliable interactions between cognitive measures and age group. When predicting Raven’s scores spatial span scores and age group, spatial span scores were a reliable predictor (*b* = 2.089, *cD* = 0.997) while the interactions between spatial span score and age were not reliable (*b* = 0.872, *cD* = 0.801). When predicting Raven’s scores with N-back scores and age group, N-back scores were a reliable predictor (*b* = 3.845, *cD* = 0.976) while the interactions between N-back score and age were not reliable (*b* = 0.538, *cD* = 0.594).

Bivariate relations between task scores (e.g., between N-back and Raven’s) are influenced by task-specific random variation; we mitigated this variation by calculating a composite score. We calculated composite scores as geometric means in order to minimize the effects of distributional differences between predictor tasks. When predicting Raven’s scores using a working memory (WM) composite score (i.e., geometric mean of N-back and spatial span scores) and age group, WM composite scores were a reliable predictor (*b* = 3.424, *cD* = 0.999) while the interactions between WM composite score and age were not reliable (*b* = 1.228, *cD* = 0.797). This composite score regression provides further support for close and developmentally-stable links between working memory and reasoning.

#### Predicting reasoning scores using measures of visual attention

**Fig 4:**
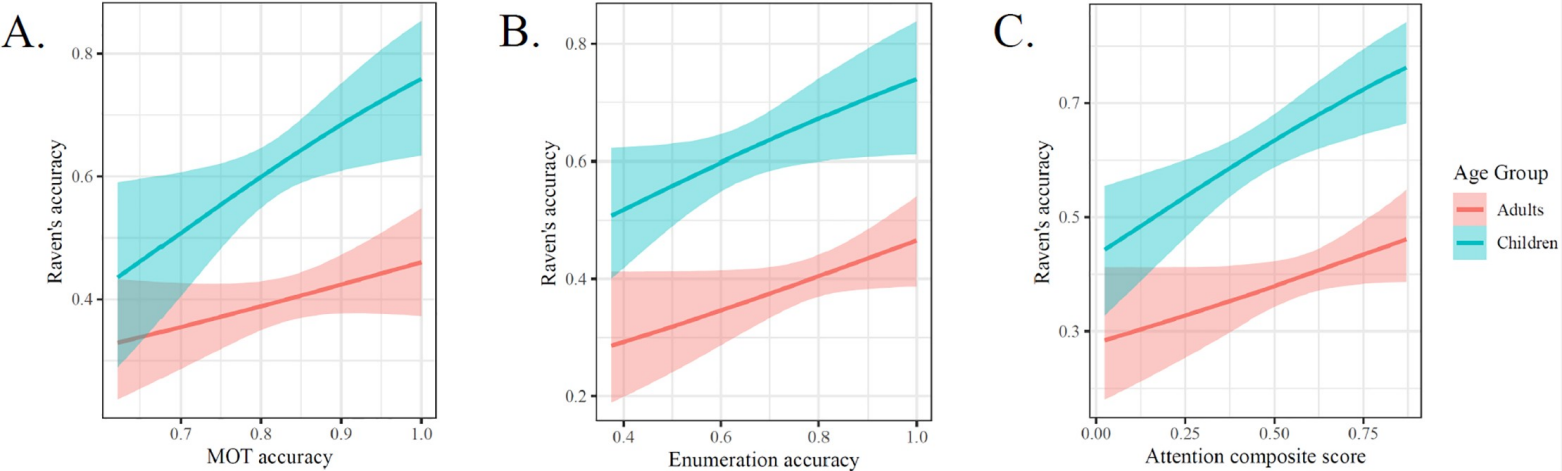
Effects of key attention measures in predicting Raven’s scores. Main effects of each attention measure were reliably predictive of reasoning scores. No interactions with age were reliable.

Next, we examined the relations between age, Raven’s scores, and attention measures (see Fig 4]. When predicting Raven’s scores using enumeration scores, and age group, enumeration scores were a reliable predictor (*b* = 1.606, *cD* = 0.979) while the interactions between enumeration score and age were not reliable (*b* = -0.375, *cD* = 0.65). When predicting Raven’s scores using MOT scores and age group, MOT scores were a reliable predictor (*b* = 3.729, *cD* = 0.991) while the interactions between MOT score and age were not reliable (*b* = -2.265, *cD* = 0.887). When predicting Raven’s scores using attention composite (i.e., geometric mean of enumeration and MOT scores) and age group, attention composite scores were a reliable predictor (*b* = 1.653, *cD* = 0.999) while the interactions between attention composite and age were not reliable (*b* = -0.737, *cD* = 0.817). As we found with working memory measures, attention is also linked to reasoning in a developmentally-stable pattern.

#### Does working memory mediate the relations between attention and reasoning?

Next we tested whether attention measures are uniquely related to Raven’s scores or whether their relations are mediated by the same capacity constraints as the working memory measures. Theories of memory and intelligence (e.g., [26]), suggest that the relations between attention and reasoning would be mediated by working memory scores. To test this hypothesis we fit Bayesian mediation models, using the same basic modeling methods described above, to estimate the degree to which relations between WM composite scores explain the effects of enumeration and MOT predicting reasoning scores. Age was included as a covariate for both expected value and precision parameters, but the interaction between age and other predictors was omitted in order to simplify model interpretation and because no interactions were evident in previous analyses.

The model parameters are reported in each of the tables below, which provide evidence that the relations between attention measures and reasoning scores are in fact mediated by a common ability captured by the WM composite scores (see Tables 3 and 4). Each of the indirect effects were reliably above zero, the proportions mediated were essentially 100%, and the direct effects of attention measures on reasoning scores was reduced to be indistinguishable from zero.

**Table 3:** Effect of enumeration on Ravens when mediated by WM composite, controlling for age-related main effects.

Effect	Estimate	HDI 2.5%	HDI 97.5%
Direct effect	-0.03	-0.96	0.93
Indirect effect	***8*.*45***	4.86	11.88
Proportion mediated	***1*.*01***	0.89	1.13

HDI indicates Highest Density Interval quantiles. Direct effect indicates the remaining effect of enumeration after accounting for the mediation of WM composite. Indirect effect indicates the strength of the mediation. Proportion mediated indicates the amount of the enumeration effect explained by the mediation.

**Table 4:** Effect of MOT on Ravens when mediated by WM composite, controlling for age-related main effects.

Effect	Estimate	HDI 2.5%	HDI 97.5%
Direct effect	0.55	-0.86	1.97
Indirect effect	***9*.*27***	4.68	13.90
Proportion mediated	***0*.*94***	0.79	1.10

HDI indicates Highest Density Interval quantiles. Direct effect indicates the remaining effect of MOT after accounting for the mediation of WM composite. Indirect effect indicates the strength of the mediation. Proportion mediated indicates the amount of the MOT effect explained by the mediation.

Importantly, the reverse mediation effect was not observed (see S3 File for full tables). The direct effects of N-back and MOT on Raven’s each remained reliable. The reliability was lower for the effect of attention composite scores mediating the relations between memory measures and Raven’s scores. The estimated proportion mediated in these models was approximately 50%. Because links between attention and reasoning were fully mediated by memory, but not the reverse, this implies a hierarchical structure of cognition and reasoning in which attentional processes underlie working memory, which in turn underlies higher-level abilities.

## Discussion

Fluid intelligence varies widely across individuals, and this variance predicts both real-world behaviors and performance on psychological tasks designed to tap core cognitive processes [15,79]. Working memory has been particularly associated with fluid intelligence [8,10,33,80]. Specifically, the controlled-attention aspect of working memory capacity has been consistently related to reasoning processes. Here we report tests of the generality of fluid intelligence abilities in children and adults, and we examine the specificity of the present relations between cognitive measures and reasoning scores.

### Developmental stability in relations between attention, memory, and fluid intelligence

In our sample of adults and children, there were no notable effects of age outside of main effects. When comparing correlations between scores on Raven’s matrices and scores on all other cognitive measures, the pattern of adults’ and children’s correlations were not different. The only difference in correlations was evident in a vocabulary measure which was correlated with reasoning in children but not adults [81]. Our findings stand in contrast to some previously published evidence for a variety of developmental relations between intelligence and cognitive demands. For instance, Cowan et al. [27] found that certain measures of children’s working memory (e.g., verbal and visual span task scores) had higher correlations with intelligence than adults’ correlations. In this same work the authors found that adults’ intelligence was better explained by the relative benefits of focused attention. It is possible that the differences between our tasks and theirs, specifically regarding our inclusion of canonical attention tasks, may have led to divergent results.

Our results, in general, point to qualitative stability in the cognitive bases of intelligence from middle childhood to early adulthood. One possible cause for developmental stability would be that fluid intelligence, by definition, is associated with a general latent factor of performance. That is, if Raven’s is a measure of fluid intelligence by this definition, then cognitive measures should generally correlate with Raven’s at any age. In stark contrast, in our data only a small number of cognitive tasks appeared to be strongly related to fluid intelligence (rather than all tasks). When examining those measures’ prediction of Raven’s in more detail, there was a consistent absence of age-related differences in parameters (i.e., null interactions).

**Fig 5:**
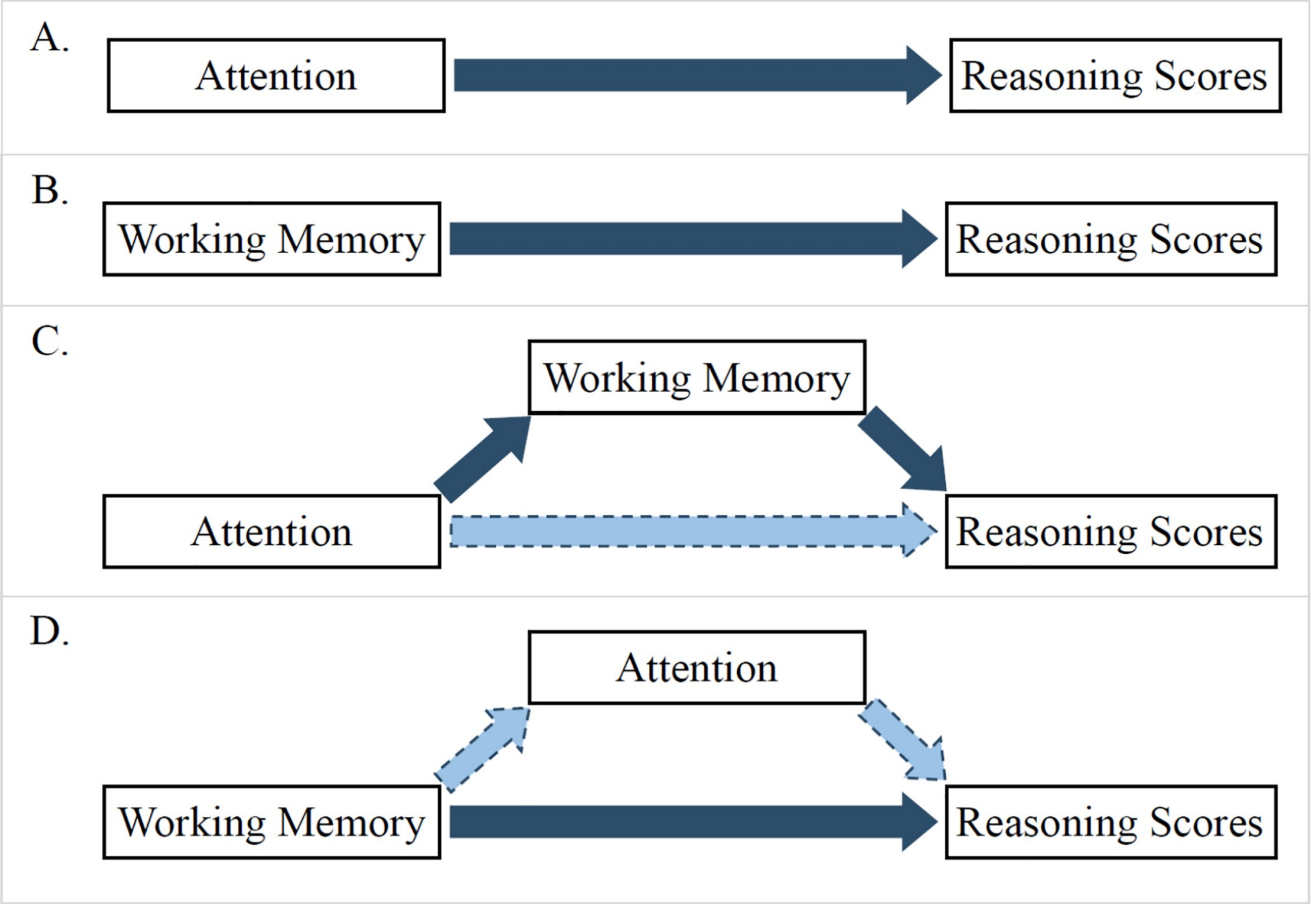
Summary of study results. Attention and working memory were each reliably associated with reasoning scores (A, B). Working memory fully mediated the effect of attention (C), but attention did not fully mediate the effect of working memory (D). Note that, because no differences in these patterns due to age were found, age-related effects are not represented in this Fig.

In the subset of cognitive tasks highly related to fluid intelligence, several were canonical measures of attention while several were measures of working memory. It was *a priori* unclear whether the shared processes giving rise to correlations between reasoning and working memory measures would be the same processes underlying correlations between reasoning measures and attention. Using mediation models we found evidence that these processes are likely to be largely overlapping; a working memory composite measure fully mediated the relations between attention measures and reasoning (see Fig 5 for a conceptual representation of this pattern).

### Relevance to experimental studies of cognition

In addition to correlational designs such as the one presented here, experimental evidence for shared processes between tasks has come from inducing a change in performance in one area and subsequently observing changes in another area. This has the potential to be a very powerful method of investigating the low-level commonalities between complex behaviors. For example, in the field of perceptual learning and generalization, tests of generalization are frequently utilized for the purpose of identifying a locus of learning-related changes [82,83]. As early as Thorndike [84], tests of generalization were used to assess the degree to which tasks’ shared properties were learned. This in turn allowed for inferences regarding the processes demanded by those tasks.

This is also the theoretical basis for cognitive training paradigms involving practice with working memory tasks that have then been linked to improvements in fluid intelligence scores (e.g., including both spatial span tasks; [73,85], and complex working memory tasks; [17,72,86]; for reviews see [16,87]). This approach has been implemented using "controlled attention" training as well, but less frequently (for a review see [88]. The body of cognitive training results is highly contentious [85,87,89–91], but these studies are well-constructed to address questions of shared processes between tasks.

It is interesting, then, that attention tasks have been used as the control group in cognitive training studies [48,92]. An alternative theoretical approach would be the assumption of a general cognitive-capacity perspective (e.g., modeling capacity-constraint theories as applicable to both attention and memory, see [31]. To the degree that attention is a necessary aspect of working memory, and has been related to fluid intelligence, there is theoretical reason to predict that training on attention-demanding tasks may lead to improvements on working memory tasks or even fluid intelligence. In fact, Thompson [92] observed a significant improvement in matrix reasoning scores after training on an adaptive MOT task, although the authors note that similar improvements were not observed in a different matrix reasoning task. Our results indicate that these results may be due to overlapping processes in MOT and matrix reasoning tasks, and that the links between attention and reasoning should be further studied. In particular, relating reasoning scores to both canonical attention tasks and canonical memory tasks may allow us to identify the underlying capacity-constraining processes that give rise to individual differences in each of these tasks. Although our results provided evidence for a common capacity-constraining process in children and adults, our conclusions would benefit from more thorough sampling of certain constructs. We were only able to address reasoning as it is measured by Raven’s scores, which is a somewhat coarse measure due to a limited number of test items. Future work would benefit from a more thorough examination of reasoning tasks themselves (e.g., [80]) as well as a wider range of these tasks.

## Conclusions

The relations between measures of attention, memory, and reasoning, which are reliable across a wide age range, provide evidence for developmental stability in the structure of cognitive processes. This stable pattern includes direct links between canonical measures of reasoning and of visual attention. The observed links are in turn mediated by working memory scores, indicating shared processes giving rise to the observed variance in each of these tasks. These mediations indicate support for a hierarchical model in which reasoning is related to attention, but only to the degree that attention supports effective memory. Despite the evidence for shared processes between reasoning and certain measures of attention and working memory, various other tasks were much less related to reasoning scores. While this may be unexpected from the perspective of fluid intelligence as a general latent factor of performance, the presence of a core set of predictors in our data indicates specificity in the cognitive bases of reasoning across developmental time. Capacity limitations in attention tasks as well as in memory tasks are closely related to fluid intelligence.

## Supporting information

S1 File Supporting Information 1. Information regarding participant exclusions.
(DOCX)

S2 File Supporting Information 2. Descriptive statistics of results.
(DOCX)

S3 File Supporting Information 3. Supplementary analyses.
(DOCX)

S1 Data (CSV)
