# Supplementary material for: Fluid intelligence is related to capacity in memory as well as attention: Evidence from middle childhood and adulthood
Source: PLoS One. 2019 Aug 22;14(8):e0221353. doi: 10.1371/journal.pone.0221353 (PMC6705795; doi:10.1371/journal.pone.0221353)
Supplement: S1 File — Information regarding participant exclusions. (DOCX) [file pone.0221353.s001.docx]

# Supporting Information 1

#### Exclusions

Exclusions were made for the following reasons. Raven’s threshold for exclusion was answering correctly on only zero or one items. For other tasks with probabilities associated with chance performance, we used an exclusion threshold set at the level that performance is significantly better than chance (i.e., one-tailed binomial test p-value less than .05). Tasks with no clearly-defined chance-performance level were tested for outliers using the method described in Leys, Ley, Klein, Bernard, and Licata (2013). No participants were excluded using this latter method.

Table S1. Exclusions.

| TASK | CRITERION | # EXCLUDED (CHILDREN) | # EXCLUDED (ADULTS) |
| --- | --- | --- | --- |
| Raven’s | < 2 correct | 0 | 10 |
| MOT | Binomial test | 2 | 2 |
| N-back | Binomial test | 0 | 1 |

*Note: No participants met exclusion criteria for tasks other than the 3 listed here*
