# Supplementary material for: Fluid intelligence is related to capacity in memory as well as attention: Evidence from middle childhood and adulthood
Source: PLoS One. 2019 Aug 22;14(8):e0221353. doi: 10.1371/journal.pone.0221353 (PMC6705795; doi:10.1371/journal.pone.0221353)
Supplement: S2 File — Descriptive statistics of results. (DOCX) [file pone.0221353.s002.docx]

# Supporting Information 2

Table S2. Means, standard deviations, and product-moment correlations

|  | 1 | 2 | 3 | 4 | 5 | 6 | 7 | 8 | 9 | 10 | 11 | 12 | 13 | 14 |
| --- | --- | --- | --- | --- | --- | --- | --- | --- | --- | --- | --- | --- | --- | --- |
| 1. UFOV |  | .38 | .22 | .06 | .38 | .23 | .15 | .15 | .15 | -.13 | -.06 | .33 | .05 | .25 |
| 2. enum. | .34 |  | -.1 | -.25 | .47 | .48 | .55 | .31 | .42 | -.18 | .08 | .2 | .22 | .27 |
| 3. conflict | -.11 | -.06 |  | .13 | -.16 | -.14 | .07 | -.17 | -.18 | .18 | -.19 | -.02 | -.02 | -.33 |
| 4. CD filter | -.11 | -.15 | .2 |  | -.22 | -.32 | -.23 | -.12 | -.13 | .01 | -.07 | .01 | 0 | -.02 |
| 5. Spat.Span | .39 | .52 | .07 | .04 |  | .35 | .38 | .41 | .35 | -.24 | -.01 | .16 | .25 | .4 |
| 6. MOT | .23 | .31 | .1 | -.13 | .34 |  | .46 | .38 | .08 | -.1 | .1 | .26 | .23 | .13 |
| 7. N-back | .25 | .42 | -.03 | -.04 | .44 | .35 |  | .3 | .3 | -.3 | .17 | .35 | .07 | -.01 |
| 8. Ravens | .09 | .21 | .03 | -.05 | .41 | .17 | .38 |  | .14 | -.3 | .07 | .33 | .24 | .6 |
| 9. PDMS | .24 | .22 | 0 | -.04 | .26 | .16 | .27 | .12 |  | -.12 | -.16 | .05 | .09 | -.03 |
| 10. CDMS | -.05 | -.13 | 0 | .14 | 0 | -.18 | -.07 | -.06 | -.25 |  | -.1 | -.02 | .12 | -.14 |
| 11. ODMS | -.26 | -.04 | .14 | -.01 | -.18 | -.1 | -.28 | -.02 | -.24 | .09 |  | .14 | -.06 | .13 |
| 12. CD | .24 | .35 | -.16 | .08 | .19 | .11 | .16 | .05 | 0 | -.11 | -.26 |  | .28 | .22 |
| 13. alert | .1 | -.04 | -.28 | -.18 | -.07 | .01 | .04 | .11 | .03 | .14 | .15 | -.04 |  | .09 |
| 14. PPVT | .17 | .08 | -.05 | .06 | .03 | -.07 | .06 | .04 | .03 | .08 | -.17 | .12 | .01 |  |
| child_means | -5.5 | .64 | .17 | .067 | .67 | .82 | .86 | .62 | .63 | 12 | 29 | .74 | .056 | .71 |
| child_SD | .69 | .13 | .18 | .14 | .14 | .062 | .052 | .16 | .098 | 3.7 | 14 | .14 | .11 | .16 |
| adult_means | -4.2 | .78 | .2 | .056 | .74 | .84 | .85 | .4 | .61 | 9.6 | 28 | .74 | .054 | .61 |
| adult_SD | .47 | .12 | .093 | .091 | .098 | .077 | .064 | .17 | .084 | 2.8 | 11 | .1 | .075 | .19 |

*Children’s correlations are listed above the diagonal, adults’ correlations below. Note that the distribution of ANT orienting scores was essentially zero-centered, and this measure was excluded for unreliability. “Conflict” and “alert” refer to the two other ANT measures, which are calculated such that larger values are better.*
