# Supplementary material for: Fluid intelligence is related to capacity in memory as well as attention: Evidence from middle childhood and adulthood
Source: PLoS One. 2019 Aug 22;14(8):e0221353. doi: 10.1371/journal.pone.0221353 (PMC6705795; doi:10.1371/journal.pone.0221353)
Supplement: S3 File — Supplementary analyses. (DOCX) [file pone.0221353.s003.docx]

# Supporting Information 3


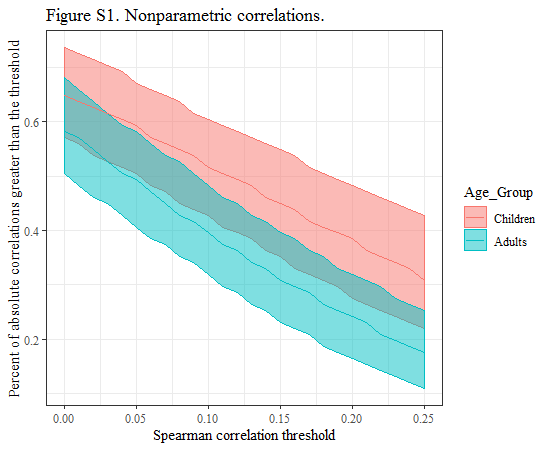


*As the threshold for identifying a “positive manifold” increased, the number of correlations that satisfy that threshold decreased. Error bands denote 95% percentiles from 10,000 bootstrapped samples for each of 26 evenly spaced thresholds.*

Table S3: Predicting Raven’s using spatial span score

|  | cD | Estimate | CI 02.5% | CI 97.5% | R-hat |
| --- | --- | --- | --- | --- | --- |
| Intercept | 0.966 | -0.919 | -1.930 | 0.0739 | 1 |
| Age | ***0.986*** | -1.690 | -3.200 | -0.2000 | 1 |
| Spat.Span | ***0.997*** | 2.090 | 0.612 | 3.5900 | 1 |
| Age × Spat.Span | 0.801 | 0.872 | -1.230 | 2.9700 | 1 |

*cD indicates the proportion of the posterior distribution with the same sign as the expected value [Estimate]. CI indicates quantiles of the posterior distribution. R-hat is a model diagnostic which, when very close to 1, indicates convergence.*

Table S4: Predicting Raven’s using N-back percent correct

|  | cD | Estimate | CI 02.5% | CI 97.5% | R-hat |
| --- | --- | --- | --- | --- | --- |
| Intercept | 0.953 | -2.840 | -6.13 | 0.494 | 1 |
| Age | 0.744 | -1.280 | -5.21 | 2.540 | 1 |
| N-back | ***0.976*** | 3.840 | -0.03 | 7.670 | 1 |
| Age × N-back | 0.594 | 0.538 | -3.92 | 5.110 | 1 |

*cD indicates the proportion of the posterior distribution with the same sign as the expected value [Estimate]. CI indicates quantiles of the posterior distribution. R-hat is a model diagnostic which, when very close to 1, indicates convergence.*

Table S5: Predicting Raven’s using a geometric mean of n-back and spatial span.

|  | cD | Estimate | CI 02.5% | CI 97.5% | R-hat |
| --- | --- | --- | --- | --- | --- |
| Intercept | ***0.992*** | -2.10 | -3.78 | -0.411 | 1.01 |
| Age | 0.952 | -1.99 | -4.33 | 0.429 | 1.01 |
| WM Comp. | ***0.999*** | 3.42 | 1.19 | 5.660 | 1.01 |
| Age × WM Comp. | 0.797 | 1.23 | -1.90 | 4.230 | 1.01 |

*cD indicates the proportion of the posterior distribution with the same sign as the expected value [Estimate]. CI indicates quantiles of the posterior distribution. R-hat is a model diagnostic which, when very close to 1, indicates convergence.*

Table S6: Predicting Raven’s using enumeration percent correct

|  | cD | Estimate | CI 02.5% | CI 97.5% | R-hat |
| --- | --- | --- | --- | --- | --- |
| Intercept | 0.871 | -0.565 | -1.5600 | 0.451 | 1.00 |
| Age | 0.869 | -0.806 | -2.2300 | 0.641 | 1.00 |
| Enumeration | ***0.979*** | 1.610 | 0.0605 | 3.160 | 1.01 |
| Age × Enum. | 0.650 | -0.375 | -2.4200 | 1.610 | 1.00 |

*cD indicates the proportion of the posterior distribution with the same sign as the expected value [Estimate]. CI indicates quantiles of the posterior distribution. R-hat is a model diagnostic which, when very close to 1, indicates convergence.*

Table S7: Predicting Raven’s using Multiple Object Tracking (MOT) percent correct

|  | cD | Estimate | CI 02.5% | CI 97.5% | R-hat |
| --- | --- | --- | --- | --- | --- |
| Intercept | 0.978 | -2.580 | -5.150 | -0.0807 | 1.00 |
| Age | 0.734 | 0.957 | -2.090 | 4.0400 | 1.00 |
| MOT | ***0.991*** | 3.730 | 0.671 | 6.8700 | 1.00 |
| Age × MOT | 0.887 | -2.260 | -6.000 | 1.4300 | 1.00 |

*cD indicates the proportion of the posterior distribution with the same sign as the expected value [Estimate]. CI indicates quantiles of the posterior distribution. R-hat is a model diagnostic which, when very close to 1, indicates convergence.*

Table S8: Predicting Raven’s using a geometric mean (i.e., “Attention Composite”) of enumeration and MOT scores

|  | cD | Estimate | l.95..CI | u.95..CI | R-hat |
| --- | --- | --- | --- | --- | --- |
| Intercept | 0.8544 | -0.2731 | -0.7799 | 0.2014 | 1.00 |
| Age | 0.9460 | -0.6741 | -1.4620 | 0.1445 | 1.01 |
| Att. Comp | ***0.9987*** | 1.6530 | 0.6316 | 2.7330 | 1.00 |
| Age× Att. Comp | 0.8168 | -0.7373 | -2.2220 | 0.6681 | 1.01 |

*cD indicates the proportion of the posterior distribution with the same sign as the expected value [Estimate]. CI indicates quantiles of the posterior distribution. R-hat is a model diagnostic which, when very close to 1, indicates convergence.*

Table S9. Effect of N-back on Ravens when mediated by attention composite, controlling for age-related differences. HDI indicates Highest Density Interval quantiles.

| Effect | Estimate | HDI 2.5% | HDI 97.5% |
| --- | --- | --- | --- |
| Direct effect | ***3.39*** | 1.50 | 5.68 |
| Indirect effect | 3.79 | -0.16 | 8.38 |
| Proportion mediated | 0.52 | 0.12 | 0.92 |

*HDI indicates Highest Density Interval quantiles. Direct effect indicates the remaining effect of N-back after accounting for the mediation of attention composite. Indirect effect indicates the strength of the mediation. Proportion mediated indicates the amount of the N-back effect explained by the mediation.*

Table S10. Effect of spatial span on Ravens when mediated by attention composite, controlling for age-related differences. HDI indicates Highest Density Interval quantiles.

| Effect | Estimate | HDI 2.5% | HDI 97.5% |
| --- | --- | --- | --- |
| Direct effect | ***2.10*** | 1.12 | 3.09 |
| Indirect effect | 1.68 | -0.28 | 3.72 |
| Proportion mediated | 0.45 | 0.07 | 0.82 |

*HDI indicates Highest Density Interval quantiles. Direct effect indicates the remaining effect of spatial span after accounting for the mediation of attention composite. Indirect effect indicates the strength of the mediation. Proportion mediated indicates the amount of the spatial span effect explained by the mediation.*

Table S11. Conventional frequentist regression results using N-back to predict Ravens scores.

|  | Estimate | t_value | p_value | boot_est | boot_t | boot_p |
| --- | --- | --- | --- | --- | --- | --- |
| N-back | 0.298 | 1.981 | 0.050 | 0.296 | 2.024 | 0.046 |
| N-back:Age | -0.052 | -0.278 | 0.781 | -0.011 | -0.074 | 0.941 |

*Predictor values were standardized within age groups, which precludes age-related main effects. “Boot” refers to mean estimates from 50,000 bootstrapped samples in which both age groups had equal numbers of cases.*

Table S12. Conventional frequentist regression results using Spatial Span to predict Ravens scores.

|  | Estimate | t_value | p_value | boot_est | boot_t | boot_p |
| --- | --- | --- | --- | --- | --- | --- |
| Spat.Span | 0.414 | 2.906 | 0.004 | 0.430 | 3.016 | 0.003 |
| Spat.Span:Age | -0.004 | -0.021 | 0.983 | -0.011 | -0.048 | 0.962 |

*Predictor values were standardized within age groups, which precludes age-related main effects. “Boot” refers to mean estimates from 50,000 bootstrapped samples in which both age groups had equal numbers of cases.*

Table S13. Conventional frequentist regression results using Enumeration to predict Ravens scores.

|  | Estimate | t_value | p_value | boot_est | boot_t | boot_p |
| --- | --- | --- | --- | --- | --- | --- |
| enum. | 0.313 | 2.073 | 0.040 | 0.319 | 2.139 | 0.035 |
| enum:Age | -0.090 | -0.484 | 0.629 | -0.101 | -0.474 | 0.637 |

*Predictor values were standardized within age groups, which precludes age-related main effects. “Boot” refers to mean estimates from 50,000 bootstrapped samples in which both age groups had equal numbers of cases.*

Table S14. Conventional frequentist regression results using MOT to predict Ravens scores.

|  | Estimate | t_value | p_value | boot_est | boot_t | boot_p |
| --- | --- | --- | --- | --- | --- | --- |
| MOT | 0.377 | 2.496 | 0.014 | 0.368 | 2.540 | 0.013 |
| MOT:Age | -0.219 | -1.179 | 0.241 | -0.201 | -0.996 | 0.322 |

*Predictor values were standardized within age groups, which precludes age-related main effects. “Boot” refers to mean estimates from 50,000 bootstrapped samples in which both age groups had equal numbers of cases.*
